# Effects of CD2-associated protein deficiency on amyloid-β in neuroblastoma cells and in an APP transgenic mouse model

**DOI:** 10.1186/s13024-015-0006-y

**Published:** 2015-03-19

**Authors:** Fan Liao, Hong Jiang, Subhashini Srivatsan, Qingli Xiao, Katheryn B Lefton, Kaoru Yamada, Thomas E Mahan, Jin-Moo Lee, Andrey S Shaw, David M Holtzman

**Affiliations:** Department of Neurology, Hope Center for Neurological Disorders, Charles F. and Joanne Knight Alzheimer’s Disease Research Center, Washington University School of Medicine, St. Louis, MO USA; Department of Pathology and Immunology, Washington University School of Medicine, St. Louis, MO USA; Department of Neurology, Hope Center for Neurological Disorders, Washington University School of Medicine, St. Louis, MO USA; Department of Neurology, Washington University School of Medicine, St. Louis, MO USA; Department of Neuropathology, Graduate School of Medicine, The University of Tokyo, Tokyo, Japan

**Keywords:** CD2AP, Alzheimer’s disease, Amyloid-β

## Abstract

**Background:**

CD2-associated protein (CD2AP) is an SH3-containing scaffold adaptor protein which regulates the actin cytoskeleton. Recently, CD2AP was identified as a genetic risk factor for Alzheimer’s disease (AD) by several genome-wide association studies. One of the hallmarks of AD is the accumulation of aggregated forms of Amyloid-β (Aβ) in the brain. In humans, CD2AP AD susceptibility locus (rs9349407) is associated with an increased plaque burden. Aβ production is highly regulated by endocytosis and is influenced by lysosomal function. Lysosomal trafficking is influenced by CD2AP. In this study, we decreased CD2AP levels in N2a neuroblastoma cultures and PS1APP mice and analyzed Aβ levels and plaque burden.

**Results:**

Our data show that suppressing CD2AP expression using shRNA in N2a-APP695 cells results in decreased cell membrane amyloid precursor protein, decreased Aβ release and a lower Aβ_42_/Aβ_40_ ratio. CD2AP protein is expressed in the brain as detected by western blot, and the expression level is dependent on gene dosage. In 1-month old PS1APP mice, complete loss of CD2AP in brain resulted in a decreased Aβ_42_/Aβ_40_ ratio in brain tissue lysates while there was no effect on Aβ deposition or accumulation in PS1APP mice expressing one copy of CD2AP.

**Conclusion:**

CD2-Associated Protein affects Aβ levels and Aβ_42_/Aβ_40_ ratio *in vitro*. The effect of CD2-Associated Protein on Aβ metabolism is subtle *in vivo*.

## Background

CD2-associated protein (CD2AP) was originally identified as a scaffold protein required for organization of the immunological synapse - the specialized interface between a T lymphocyte and an antigen-presenting cell [1]. It was later shown that CD2AP, by virtue of its multiple protein-protein binding modules, interacts with multiple proteins involved in diverse biological processes. These associations have implicated CD2AP in receptor tyrosine kinase internalization, actin cytoskeleton remodeling and vesicular trafficking [2].

Alzheimer’s disease (AD) is a neurodegenerative disorder characterized by the impairment of memory and other cognitive functions as well as the presence of extracellular amyloid plaques and intracellular neurofibrillary tangles [3]. There is substantial evidence indicating that amyloid-β (Aβ) plays an essential role in the development of AD [4,5]. The deposition of Aβ into amyloid plaques is dependent on the concentration of brain interstitial fluid Aβ [6], which is regulated by endocytosis [7]. Recently CD2AP was detected as a risk factor for AD by several genome-wide association studies [8-10]. In a yeast model for cellular toxicity elicited by Aβ, a functional homolog of CD2AP [11] was identified as a suppressor of Aβ toxicity [12]. In addition, a recent study in humans suggests that CD2AP AD susceptibility locus (rs9349407) is associated with increased plaque burden [13]. However, the relationship between CD2AP and Aβ has never been reported in mammalian cells or in mouse models expressing human amyloid precursor protein (APP)/Aβ.

Since the reported AD susceptibility locus (rs9349407) which has impact on plaque load in humans [13] is in the CD2AP gene, we asked whether manipulating CD2AP expression level affects Aβ levels. In the current study, we knocked down CD2AP expression in N2a-APP695 cells and observed a decrease in Aβ levels as well as the ratio of Aβ_42_/Aβ_40_ in the cell culture medium. We crossed CD2AP knockout mice with PS1APP mice and observed a reduction of Aβ_42_/Aβ_40_ in the brain tissue. Due to the fact that CD2AP knockout mice have glomerular disease and do not survive beyond a few months of age until plaque onset in PS1APP mice [14], we also measured the effects of CD2AP haploinsufficiency on amyloid plaque deposition. There was no effect of CD2AP haploinsufficiency on Aβ deposition up to 7 months of age.

## Results and discussion

### Effects of CD2AP deficiency in cultured N2a-APP695 cells

**Figure 1:**
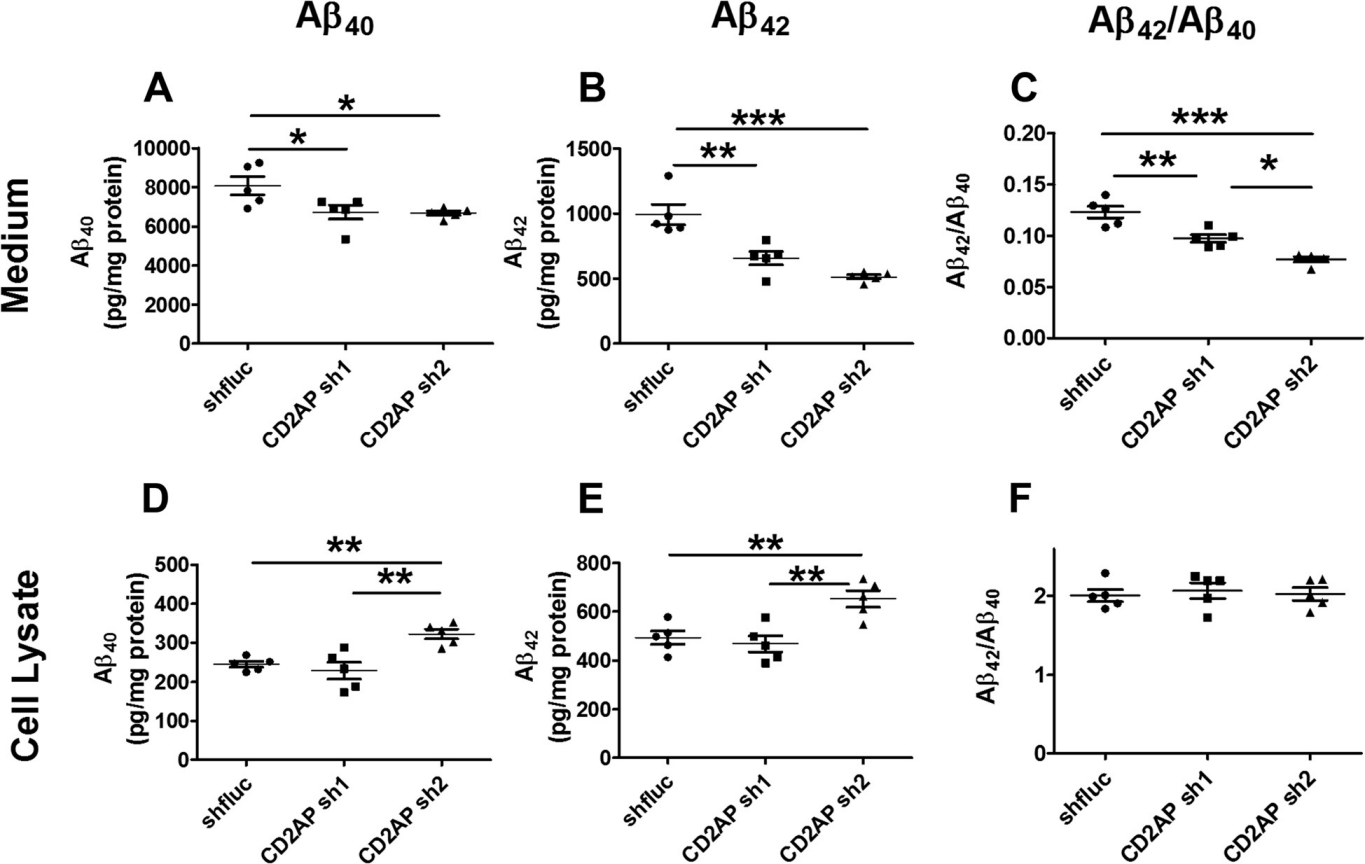
**CD2AP shRNAs decreased extracellular Aβ levels in cell culture.** N2a-695 cells were transfected with CD2AP shRNAs or control shRNA. **(A-** **C)** Aβ_40_, Aβ_42_ and Aβ_42_/Aβ_40_ ratio in the cell culture medium. **(D-** **F)** Aβ_40_, Aβ_42_ and Aβ_42_/Aβ_40_ ratio in the cell lysates (n = 5/group; *, *p* < 0.05, **, *p* < 0.01, ***, *p* < 0.001, one-way ANOVA followed by Tukey test).

**Figure 2:**
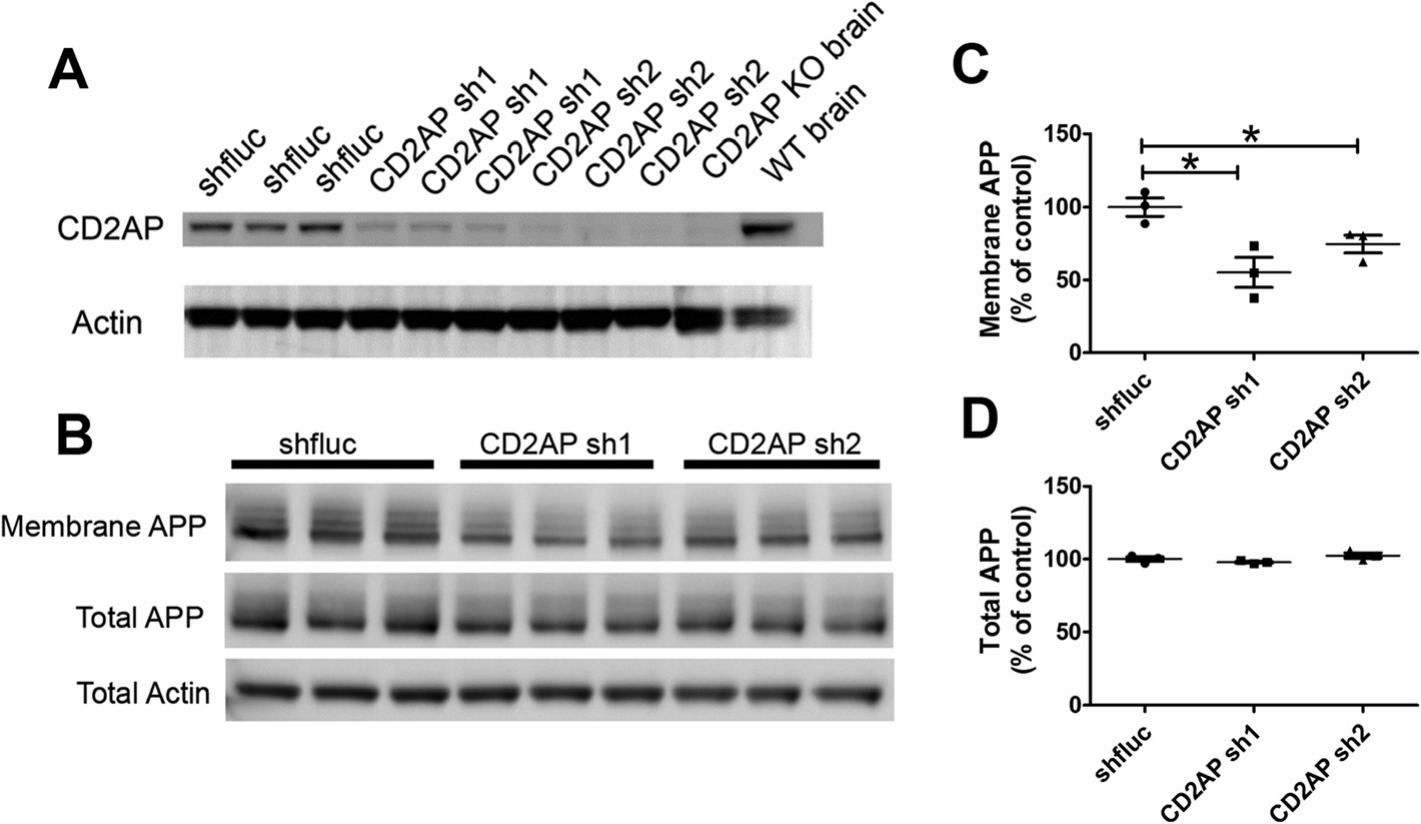
**CD2AP shRNAs decreased cell membrane APP. (A)** CD2AP and actin levels in cells transfected with CD2AP shRNAs or control shRNA. **(B)** Membrane and total APP levels in the cells transfected with CD2AP shRNAs or control shRNA. **(C-** **D)** Quantification of membrane APP and total APP in H (n = 3/group, *, *p* < 0.05, Student’s *t*-test).

APP processing is regulated by endocytosis. Given that CD2AP plays an important role in regulating endocytosis, we first tested whether CD2AP has an effect on Aβ synthesis or Aβ release in cultured cells. We used CD2AP shRNA to knockdown CD2AP levels (Figures 1, 2A) in neuroblastoma N2a-APP 695 cells and measured Aβ in the cell lysates and Aβ released into the cell culture medium. The results showed that CD2AP sh RNA1 (Sh1) significantly decreased both Aβ_40_ and Aβ_42_ secreted into the culture medium by about 20 ~ 30% (Figure 1A,B) while the Aβ_40_ and Aβ_42_ in cell lysates were not affected (Figure 1D,E). CD2AP sh2 had greater effects on the Aβ_42_ in the medium as compared to CD2AP sh1 (Figure 1A,B). The Aβ_40_ and Aβ_42_ in cell lysates were increased about 30% by CD2AP sh2 (Figure 1D,E). Interestingly, both CD2AP sh1 and CD2AP sh2 decreased the Aβ_42_/ Aβ_40_ ratio in cell culture medium (Figure 1C). However, the Aβ_42_/ Aβ_40_ ratios in cell lysates were unaltered (Figure 1F). We further examined the total APP and APP on the cell surface in these N2a cells. The results showed that CD2AP sh1 and sh2 did not change total APP levels in N2a cells (Figure 2B,D). However, membrane APP levels were decreased by CD2AP sh1 and sh2 (Figure 2B,C). In cells, nascent APP is post-translationally modified and transported from the endoplasmic reticulum to the plasma membrane [15]. To be proteolytically cleaved into Aβ, APP must be internalized from the cell surface into the cell and transported to endosomes where β-secretase and γ-secretase complexes cleave APP to produce Aβ [7,16]. In the current study, knocking down CD2AP in N2a-APP695 cells may decrease APP on cell surface which would result in less APP getting into endosomes and less Aβ being released into cell culture medium.

### Expression of CD2AP in the brain

**Figure 3:**
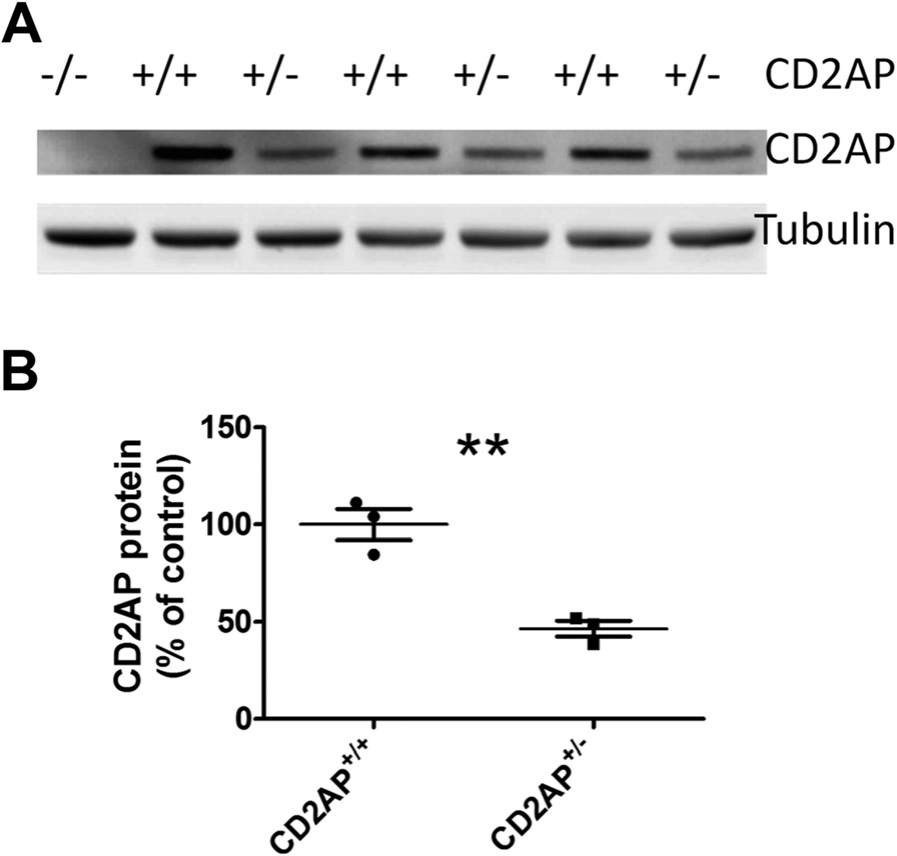
**CD2AP protein was expressed in the brain. (A)** Western blot for CD2AP and tubulin in cortices from CD2AP^−/−^, CD2AP^+/−^ and CD2AP^+/+^ mice. **(B)** Quantification of CD2AP levels in CD2AP^+/−^ and CD2AP^+/+^ mice in western blot presented in A. (n = 3/group, **, *p* < 0.01, Student’s *t*-test).

Before we studied the effects of CD2AP on Aβ pathology *in vivo*, we first determined whether CD2AP is expressed in the brain and whether the expression level correlates with CD2AP gene dosage. Using western blot, we detected CD2AP in CD2AP^+/+^ brains with CD2AP^−/−^ brains serving as a negative control (Figure 3A). As reported in the kidney [17], CD2AP protein level in the brains of mice with CD2AP haploinsufficiency (CD2AP^+/−^) is about 50% of the level in CD2AP^+/+^ mice (Figure 3B).

### Effects of CD2AP knockout on brain Aβ levels in 1-month old PS1APP mice

**Figure 4:**
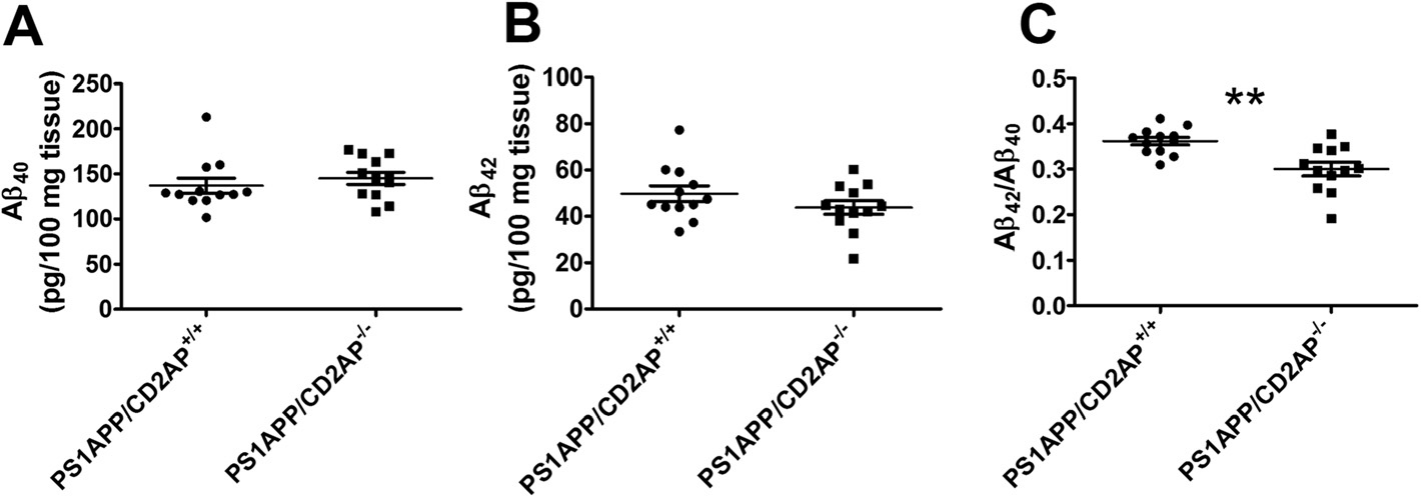
**Effects of CD2AP on Aβ levels in 1-month old PS1APP mice.** PS1APP/CD2AP^+/+^ (n = 12 total, 6 females and 6 males) and PS1APP/CD2AP^−/−^ (n = 12 total, 7 females and 5 males) mice were sacrificed at 1-month of age. The cortices were homogenized in PBS. Aβ_40_ **(A)**, Aβ_42_ **(B)** and Aβ_42_/ Aβ_40_ **(C)** ratio were measured. (**, *p* < 0.01, Student’s *t*-test).

Next, we assessed whether CD2AP has similar effects on Aβ levels and Aβ_42_/Aβ_40_ ratio *in vivo*. If CD2AP affects Aβ production or release, we would expect to see the changes in young mice before plaque deposition. Therefore, we generated 1-month old PS1APP/CD2AP^+/+^ (female, n = 6; male, n = 6) and PS1APP/CD2AP^−/−^ (female, n = 7; male, n = 5) mice and measured Aβ_40_ and Aβ_42_ levels in the PBS soluble fraction of cortical tissue. The results showed that there were no significant changes in the absolute concentration of Aβ_40_ and Aβ_42_ (Figure 4A,B). However, the ratio of Aβ_42_/Aβ_40_ was lower in PS1APP/CD2AP^−/−^ mice as compared to PS1APP/CD2AP^+/+^ mice (Figure 4C). This effect was similar in both females (0.37 ± 0.008 *vs* 0.29 ± 0.0025 for PS1APP/CD2AP^+/+^*vs* PS1APP/CD2AP^−/−^, respectively, *p* < 0.05, Student’s *t*-test) and males (0.35 ± 0.015 *vs* 0.30 ± 0.010 for PS1APP/CD2AP^+/+^*vs* PS1APP/CD2AP^−/−^, respectively, *p* < 0.05, Student’s *t*-test).

Although inhibiting CD2AP expression levels in cultured cells resulted in decreased levels of Aβ in the cell culture medium, we did not observe changes in the absolute concentration of cortical Aβ_42_ or Aβ_40_ in CD2AP knockout PS1APP mice as compared to PS1APP mice expressing two copies of CD2AP. While Aβ levels in cultured APP-expressing N2a-APP695 cells are mainly determined by APP processing, Aβ levels *in vivo* are regulated not only by Aβ production and release but also Aβ uptake and degradation by different cell types, Aβ clearance mediated by interstitial fluid (ISF) and cerebrospinal fluid (CSF) bulk flow and Aβ transport across the blood brain barrier. Among these mechanisms, many of them are regulated by vesicular trafficking, which can be influenced by manipulating CD2AP levels. For example, disruption of endocytosis in neurons inhibits APP processing and reduces Aβ levels [7]. On the other hand, disruption of endocytosis in the microglia or astrocytes could result in an increase of extracellular Aβ due to decreased Aβ uptake and degradation [18]. Therefore, these effects could theoretically cancel each other out when knockout of CD2AP occurs in all the cell types. This may explain why we did not observe the same change in Aβ concentration *in vivo* as we have seen *in vitro*.

In cultured N2a-APP695 cells, knocking down CD2AP resulted in a lower Aβ_42_/Aβ_40_ ratio. Similar changes also occurred in the soluble Aβ_42_/Aβ_40_ ratio in 1-month old PS1APP/CD2AP^−/−^ mice as compared to PS1APP/CD2AP^+/+^ mice. The ratio of Aβ_42_/Aβ_40_ is determined by γ-secretase cleavage of APP. Mutations of presenilin, an active enzymatic component of the γ-secretase complex, lead to autosomal dominant familial AD [19,20] likely in large part due to an increased Aβ_42_/Aβ_40_ ratio. On the other hand, mutations in APP [21] or gamma secretase modulators [22] can influence γ-secretase cleavage and alter the Aβ_42_/Aβ_40_ ratio. Some molecules such as phosphatidylinositol clathrin assembly lymphoid-myeloid leukemia (PICALM) shift the Aβ_42_/Aβ_40_ ratio through affecting internalization of γ-secretase [23]. In the current study, the change of Aβ_42_/Aβ_40_ ratio occurred before plaque deposition, suggesting CD2AP knockout could affect the selective production of Aβ_40_ and Aβ_42_. Since CD2AP binds membrane proteins, it could affect Aβ cleavage by interacting with γ-secretase complex, by modifying APP directly or through some intermediary molecules to shift the ratio of Aβ_42_/Aβ_40_. It is shown recently that a homolog of Nephrin, a protein interacting with CD2AP [14], is required for γ-secretase mediated Notch and APP-like cleavages in Drosophila [24].

### Effects of CD2AP haploinsufficiency on amyloid deposition in 7-month old PS1APP mice

**Figure 5:**
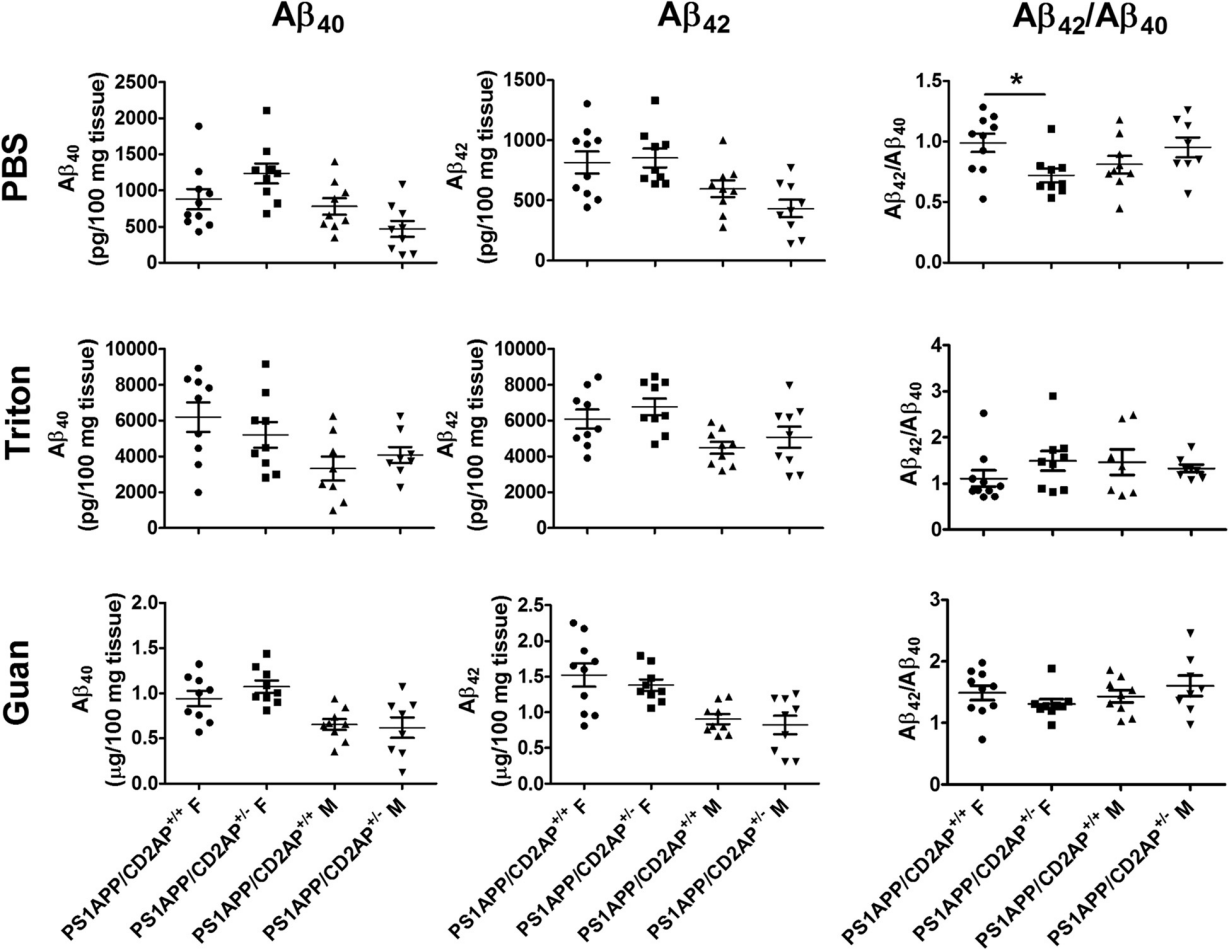
**Effects of CD2AP haploinsufficiency on tissue Aβ levels in 7-month old PS1APP mice.** PS1APP/CD2AP^+/+^ (n = 19 total, 10 females and 9 males) and PS1APP/CD2AP^+/−^ (n = 18 total, 9 females and 9 males) mice were sacrificed. Aβ_40_, Aβ_42_ and Aβ_42_/Aβ_40_ ratio in PBS fraction, 1% triton fraction and Guanidine fraction (*, *p* < 0.05, Student’s *t*-test).

Shifting the ratio of Aβ_42_/Aβ_40_ results in an altered time course of plaque deposition in both humans [19,20] and mice [25]. Since we observed changes in the Aβ_42_/Aβ_40_ ratio in cultured cells and 1-month old PS1APP mice, we next asked whether CD2AP deficiency affects amyloid plaque load in older PS1APP mice. CD2AP^−/−^ mice have a ~ 6-week life-span due to renal failure [14] while the average plaque onset age in PS1APP mice is at ~4-month. Therefore, we were not able to assess plaque deposition in PS1APP/CD2AP^−/−^ mice. Mice with CD2AP haploinsufficiency (CD2AP^+/−^) live a normal life span but express ~50% less CD2AP in the brain compared to CD2AP^+/+^ mice (Figure 3A,B). We therefore asked whether a ~50% reduction of CD2AP levels affects Aβ pathology in PS1APP mice. We generated PS1APP/CD2AP^+/−^ and PS1APP/CD2AP^+/+^ mice and characterized their Aβ pathology at the age of 7-months. We first measured Aβ levels in the cortical tissue lysates. The results showed no difference in the absolute level of Aβ_40_ and Aβ_42_ in the PBS (soluble forms of Aβ), Triton or Guanidine (insoluble forms of Aβ) brain fractions between PS1APP/CD2AP^+/−^ and PS1APP/CD2AP^+/+^ groups (Figure 5). In the PBS soluble fraction, the Aβ_42_/Aβ_40_ ratio in the female PS1APP/CD2AP^+/−^ group was significantly lower than that in female PS1APP/CD2AP^+/+^ group (*p* < 0.05, Student’s *t*-test). However, the effects on males tended to trend in the opposite direction (Figure 5). In the Triton and Guanidine fractions, there was no change in the Aβ_42_/Aβ_40_ ratio associated with CD2AP gene status (Figure 5).

**Figure 6:**
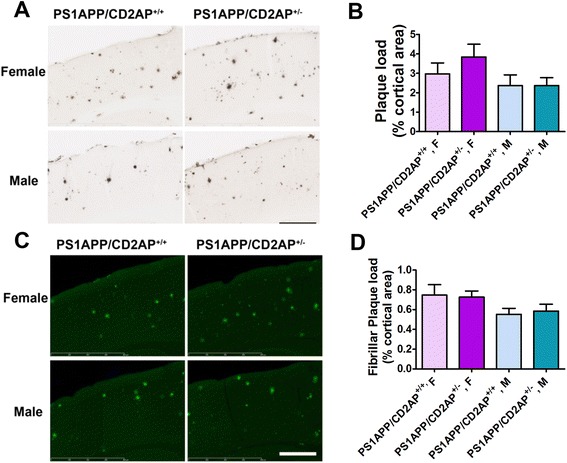
**Effects of CD2AP haploinsufficiency on Aβ plaque deposition in 7-month old PS1APP mice.** PS1APP/CD2AP^+/+^ (n = 19 total, 10 females and 9 males) and PS1APP/CD2AP^+/−^ (n = 18 total, 9 females and 9 males) mice were sacrificed. **(A)** Representative images for Aβ staining in PS1APP/CD2AP^+/+^ and PS1APP/CD2AP^+/−^ cortex (Scale bar =400 μm). **(B)** Quantification of Aβ plaque load in PS1APP/CD2AP^+/+^ and PS1APP/CD2AP^+/−^ cortex. **(C)** Representative images of Thioflavin S staining in PS1APP/CD2AP^+/+^ and PS1APP/CD2AP^+/−^ cortex (Scale bar =400 μm). **(D)** Quantification of fibrillar plaque load in PS1APP/CD2AP^+/+^ and PS1APP/CD2AP^+/−^ cortex.

To determine whether CD2AP haploinsufficiency affects the morphology or other properties of the Aβ plaques, we stained the tissue with biotinylated anti-Aβ_1–13_ monoclonal antibody HJ3.4B (Figure 6A) or Thioflavin S (Figure 6C) which stains fibrillar forms of Aβ plaques. We did not observe any significant changes in plaque distribution, individual plaque size or plaque morphology associated with CD2AP gene status. We further quantified the% area covered with plaques in the cortex. The results demonstrated that neither Aβ immunostained plaques (Figure 6B) nor fibrillar plaques (Figure 6D) were different in mice with CD2AP haploinsufficiency. Taken together, although CD2AP haploinsufficiency lowered the Aβ_42_/Aβ_40_ ratio in PBS fraction of female PS1APP mice, the majority of our data demonstrate that CD2AP haploinsufficiency did not cause changes in Aβ accumulation.

For certain genes that have clear effects on Aβ metabolism and deposition such as apolipoprotein E or PICALM, a 50% reduction of expression has significant effects on Aβ pathology [26,27]. However, in our *in vivo* studies, lowering of CD2AP expression levels by 50% in the PS1APP/CD2AP^+/−^ mice did not affect Aβ pathology as assessed by biochemical or histological methods as compared to that in control PS1APP/CD2AP^+/+^ mice at the age of 7-months. It is possible that expression of 50% the level of CD2AP is sufficient to maintain adequate CD2AP function in the brain. In the future, additional work on CD2AP conditional knockout mice which live a longer life-span can be done to verify the effects of CD2AP on amyloid deposition in mice expressing lower than levels found in C2DAP haploinsufficiency in the brain.

## Conclusions

In summary, we demonstrated that knocking down CD2AP in cultured N2A-APP695 cells reduces Aβ_40_, Aβ_42_ and the ratio of Aβ_42_/Aβ_40_ released into cell culture medium. CD2AP expression is readily detectable in the brain and so we extended out *in vitro* studies to an APP mouse model. In 1-month old PS1APP mice, a complete loss of CD2AP reduced the Aβ_42_/Aβ_40_ ratio in the cortical tissue but the absolute levels of Aβ_40_ and Aβ_42_ were unaltered. In 7-month old PS1APP mice, CD2AP haploinsufficiency did not cause significant changes in Aβ pathology as analyzed by biochemical and histological assays. In the future, conditional knockout of CD2AP in different brain cells can be produced to further confirm or refute the effects of CD2AP on Aβ pathology. Primary neurons cultured from PS1APP/CD2AP^−/−^ mice need to be used to confirm the *in vitro* effects of CD2AP on Aβ production and release. Besides Aβ plaques, another hallmark of AD is the accumulation of insoluble tau protein in structures such as intracellular neurofibrillary tangles. It was found that RNAi targeting *Cindr*, the fly ortholog of the human CD2AP, enhances tau toxicity in a Drosophila model in a recent study [28]. Therefore, CD2AP could also modify AD status by interacting with tau or potentially via other mechanisms. In the future, additional possible mechanisms should be explored to establish the mechanism(s) underlying the role of CD2AP in AD pathogenesis.

## Methods

### Animals

APPswe/PS1ΔE9 (PS1APP) mice overexpressing a chimeric mouse/human APP695 Swedish gene and human PSEN1 with an exon 9 deletion on a B6C3 background [25] were crossed with CD2AP^+/−^ mice on B6 background [14] to generate PS1APP/CD2AP^+/−^ and PS1APP/CD2AP^+/+^ (control) mice. To generate PS1APP/CD2AP^−/−^ and PS1APP/CD2AP^+/+^ (control) mice, the PS1APP/CD2AP^+/−^ mice were crossed with CD2AP^+/−^ /nephrin Tg mice. The nephrin Tg mice express CD2AP driven by mouse nephrin promoter which directs expression specifically in podocytes [29]. On the day of harvesting, the mice were perfused with ice-cold PBS containing 0.3% heparin. For the 7-month old PS1APP/CD2AP^+/−^ and PS1APP/CD2AP^+/+^ mice, one hemibrain was fixed in 4% paraformaldehyde for immunohistochemistry. The other hemibrain was dissected and flash-frozen on dry ice for biochemical assays. For all other animals, both sides of the brain were dissected for biochemical assays. All experimental protocols were approved by the Animal Studies Committee at Washington University.

### CD2AP knockdown in cultured cells

N2a-APP695 cells were grown in DMEM/Opti-MEM (50:50) supplemented with 5% FBS and 200 μg/ml of G418. Control (firefly luciferase target sequence, fLuc; GCTTACGCTGAGTACTTCGA) and two different CD2AP-specific shRNA duplexes (CD2AP target sequences, CD2AP sh1 and sh2: GTGGAACCCTGAACAATAAG and GGAACCAATGAAGATGAACTTACA, respectively) were cloned into the pFLRu lentivirus as previously described [30]. Viral supernatants were generated in 293 T cells by transfection of the lentiviral plasmids with Lipofectamine 2000 (Invitrogen) and the packaging plasmids as described. Pooled supernatants, harvested at 24 and 48 hours post-transfection, were applied to N2a cells with 8ug/ml polybrene and spun at room temperature for 2 hours at 2000 rpm. Supernatants were replaced with fresh medium immediately after centrifugation. To confirm the knock down of CD2AP, the level of CD2AP protein in RIPA cell lysates was assessed by western blot using a rabbit anti-CD2AP polyclonal antibody [1]. Actin was detected using a mouse monoclonal antibody (Sigma) which served as internal control. The cells were then cultured in a 12-well plate in serum free medium at a density of 80%. After 8 hrs, the cell culture medium was collected and the cells were homogenized in RIPA buffer. The Aβ_40_ and Aβ_42_ in the cell culture medium and cell lysates were measured by sandwich ELISA. All the values were normalized to the protein concentration in the cell lysate to correct for the differences caused by different cell numbers in each well. To assess total APP and membrane APP, N2a-fluc, N2a-sh1 and N2a sh2 cells were cultured in a 6-well plate at a density of 90%. The membrane proteins were biotinylated by incubating the cells with EZ-Link Sulfo-NHS-SS-Biotin (Thermo Scientific) on ice for 30 min. Then the cells were homogenized in lysate buffer containing 50 mM Tris–HCl, pH 7.5, 150 mM NaCl, 1% (v/v) Nonidet P-40, 0.5% (w/v) deoxycholate, and 1× protease inhibitor mixture [27]. Total APP in the cell lysates was assessed using western blot with a rabbit anti-APP polyclonal antibody (Zymed). The value was normalized to actin in the same lysates. Cell lysates containing same amount of total protein were incubated with Dynabeads® MyOne™ Streptavidin T1 (Invitrogen) to pull down biotinylated membrane protein. Western blot for APP was performed on the pull-down products to assess the membrane APP.

### Western blot for CD2AP in brain lysates

Four-week old CD2AP^+/+^, CD2AP^+/−^ and CD2AP^−/−^ mice were perfused with ice-cold PBS containing 0.3% heparin. The cortices were dissected and homogenized in RIPA buffer. CD2AP was detected by western blot using a rabbit anti-CD2AP polyclonal antibody [1]. The western blot bands were quantified using image J (National Institutes of Health). The levels of CD2AP were normalized using tubulin values from the same sample.

### ELISA

For 1-month old PS1APP/CD2AP^−/−^ and PS1APP/CD2AP^+/+^ (control) mice, brain cortices were homogenized in PBS in the presence of 1× protease inhibitor mixture (Roche). For 7-month old PS1APP/CD2AP^+/−^ and PS1APP/CD2AP^+/+^ mice, brain cortices were sequentially homogenized with cold PBS, 1% Triton, and 5 M guanidine buffer in the presence of 1× protease inhibitor mixture. The levels of Aβ_40_ and Aβ_42_ were measured by sandwich ELISA. For Aβ_40_ or Aβ_42_, anti-Aβ_35–40_ HJ2 or anti-Aβ_37–42_ HJ7.4 were used as capture antibodies, and anti-Aβ_13–28_ HJ5.1-biotin [6] was used as detecting antibody.

### Immunohistochemistry

Serial coronal sections at 50-μm thickness were collected from the rostral to the caudal end of each brain hemisphere using a freezing sliding microtome (Leica). Aβ plaques were immunostained using biotinylated anti-Aβ_1–13_ monoclonal antibody HJ3.4B [31].

### Thioflavine S staining

For fibrillar plaques, brain sections were stained with 0.025% Thioflavin S (Sigma) in 50% ethanol for 10 min. Then the sections were washed with 50% ethanol twice followed by PBS [32].

### Imaging

Immunostained brain sections were scanned using a Nanozoomer slide scanner (Hamamatsu Photonics). Quantitative analysis of immunopositive staining was performed as previously described [33]. Briefly, images of immunostained sections were exported with NDP viewer (Hamamatsu Photonics), converted to 8-bit grayscale using ACDSee Pro 2 software (ACD Systems) and threshold was set to highlight positive staining and analyzed using ImageJ (National Institutes of Health). 3 sections per mouse (Bregma, −1.4 mm caudal to Bregma, −2.0 mm caudal to Bregma) were quantified (the cortex immediately dorsal to the hippocampus) and the average was used to represent each mouse.

### Statistics

Two-tailed Student’s *t*-test was used to determine if there were significant differences between two groups unless otherwise specified. One-way ANOVA was used to compare differences among 3 or more groups followed by Tukey post-test unless otherwise specified. Data in all the figures are expressed as mean ± S.E.M unless otherwise specified.

## Abbreviations

CD2AP: CD2-Associated protein
Aβ: Amyloid-β
AD: Alzheimer’s disease
SH3KBP1: SH3-domain kinase binding protein 1
PICALM: Phosphatidylinositol clathrin assembly lymphoid-myeloid leukemia
